# Non-Invasive Bleaching of the Human Lens by Femtosecond Laser Photolysis

**DOI:** 10.1371/journal.pone.0009711

**Published:** 2010-03-16

**Authors:** Line Kessel, Lars Eskildsen, Mike van der Poel, Michael Larsen

**Affiliations:** 1 Department of Ophthalmology, Glostrup Hospital, University of Copenhagen, Glostrup, Denmark; 2 Department of Photonics Engineering, Technical University of Denmark, Kgs. Lyngby, Denmark; University of Crete, Greece

## Abstract

**Background:**

Globally, cataract is the leading cause of blindness and impaired vision. Cataract surgery is an attractive treatment option but it remains unavailable in sufficient quantity for the vast majority of the world population living in areas without access to specialized health care. Reducing blindness from cataract requires solutions that can be applied outside operating theatres. Cataract is a protein conformational disease characterized by accumulation of light absorbing, fluorescent and scattering protein aggregates. The aim of the study was to investigate whether these compounds were susceptible to photobleaching by a non-invasive procedure and whether this would lead to optical rejuvenation of the lens.

**Methodology/Principal Findings:**

Nine human donor lenses were treated with an 800 nm infra-red femtosecond pulsed laser in a treatment zone measuring 1×1×0.52 mm. After laser treatment the age-induced yellow discoloration of the lens was markedly reduced and the transmission of light was increased corresponding to an optical rejuvenation of 3 to 7 years.

**Conclusions/Significance:**

The results demonstrate that the age-induced yellowing of the human lens can be bleached by a non-invasive procedure based on femtosecond laser photolysis. Cataract is a disease associated with old age. At the current technological stage, lens aging is delayed but with a treatment covering the entire lens volume complete optical rejuvenation is expected. Thus, femtosecond photolysis has the potential clinical value of replacing invasive cataract surgery by a non-invasive treatment modality that can be placed in mobile units, thus breaking down many of the barriers impeding access to treatment in remote and poor regions of the world.

## Introduction

Cataract is the world-leading cause of blindness and visual impairment accountable for almost 20 million cases of bilateral blindness [1]. Blindness from cataract could be prevented if only high quality and high volume cataract surgery was available all over the globe [2]. This is unfortunately not the case and the barriers impeding access to treatment includes lack of skilled and well-trained surgeons, lack of sterile operating facilities, lack of the high-tech equipment required for the surgical procedure, and the high cost of the procedure rendering the surgery inaccessible to a vast majority of world population. At present there is no known ways of preventing cataract formation or any non-surgical treatment modalities.

Cataract is a protein conformational disease caused by posttranslational modifications of the structural lens proteins [3]. The human lens is a highly specialized avascular tissue with little or no turn-over of its constituent structural proteins [4]. The orderly arrangement of these proteins is essential for the transparency of the lens [5]–[8] and for quality of vision. With time the structural lens proteins are subjected to posttranslational modifications by non-enzymatic glycation [9] and other biochemical and biophysical pathways [3] leading to an accumulation of chromophores that absorb visible light preferentially in the blue end of the spectrum [10], giving the aged human lens its characteristic yellow-brown color. Additional processes of optical degradation of the aging and cataractous lens include protein aggregation leading to increased scattering of light [11]. The end result is the milky white appearance that prompted the ancient Greeks to name the condition after their word for waterfall “katarrhaktes”.

Photoactive molecules, such as chromophores, will eventually degrade upon the interaction with light. Chromophore bleaching is a phenomenon of fundamental importance for multiple life functions such as vision that is based on the photobleaching of photopigments, opsins, in the retina [12]. The fluorescent chromophores of the human lens can be bleached in vitro by broad-band ultraviolet radiation [13], [14] demonstrating that lenses can be optically rejuvenated by chromophore bleaching. Ultraviolet treatment of the lens is, however, not possible in a clinical setting because of retinal phototoxicity [15], [16].

Instead, we chose to explore a multiphoton approach where ultraviolet-like effects are obtained in confined space using infra-red light that is less harmful to the retina [17]. Multiphoton effects occur when two or more photons hit a target molecule simultaneously. In practice, this requires a very high photon density that can only be obtained by using ultra-short pulsed lasers. The chosen methodology is analogue to the bleaching of artificial fluorescent dyes known by researchers in the field of multiphoton microscopy [18]. We demonstrate that a clinically relevant level of photobleaching can be obtained in a manner that is predicted to be safe for the rest of the eye.

## Results

**Figure 1:**
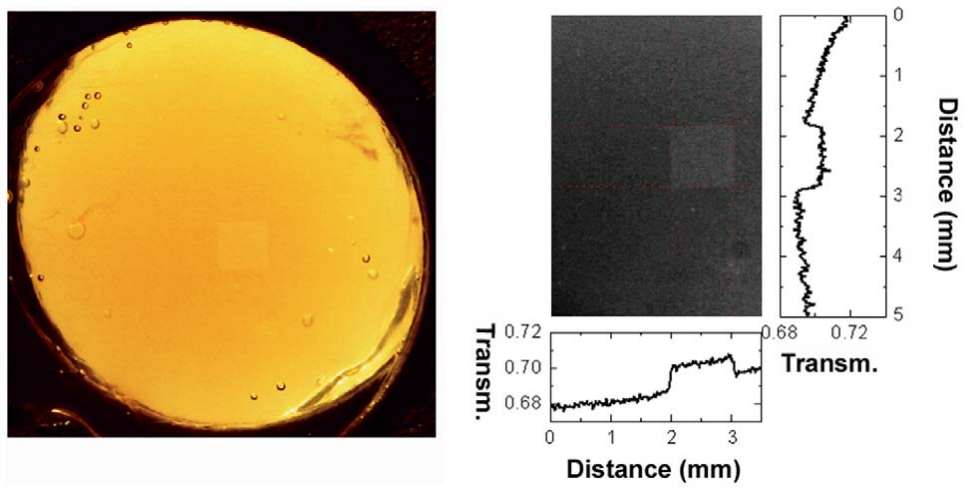
Photographic documentation of the laser photobleaching. To the right is shown a color photograph of a 58 year old donor lens after treatment and to the left is shown a black-and-white excerpt of the same photograph with an image analysis of the light intensity in the photograph. The treated area is seen as a lighter square and it is marked by red dotted lines on the black and white photograph. The relative transmission was calculated by using the black plastic spacer surrounding the lens as a reference for 0% transmission and the vacant space between the lens and the plastic spacer as a reference for 100% transmission.

Nine human organ donor lenses aged 58 to 75 years of age were exposed to infra-red 800 nm radiation from a femtosecond pulsed Ti:Sapphire laser using pulse energies ranging from 0.10 to 0.55 µJ. The laser beam was scanned across the lens in a line-by-line raster pattern covering a volume of 1mm in height ×1 mm in width ×0.52 mm in depth. Photobleaching was visible to the unaided observer in the treated area with no signs of damage to the untreated parts of the lens (Fig. 1). Using a slit-lamp biomicroscope it was seen that photobleaching was confined to the subvolume inside the lens targeted by the scanning procedure. The effect of the laser treatment remained visible 1 to 2 weeks after the treatment. Continued observation was not possible due to the gradual optical deterioration of the donor lenses post mortem.

**Figure 2:**
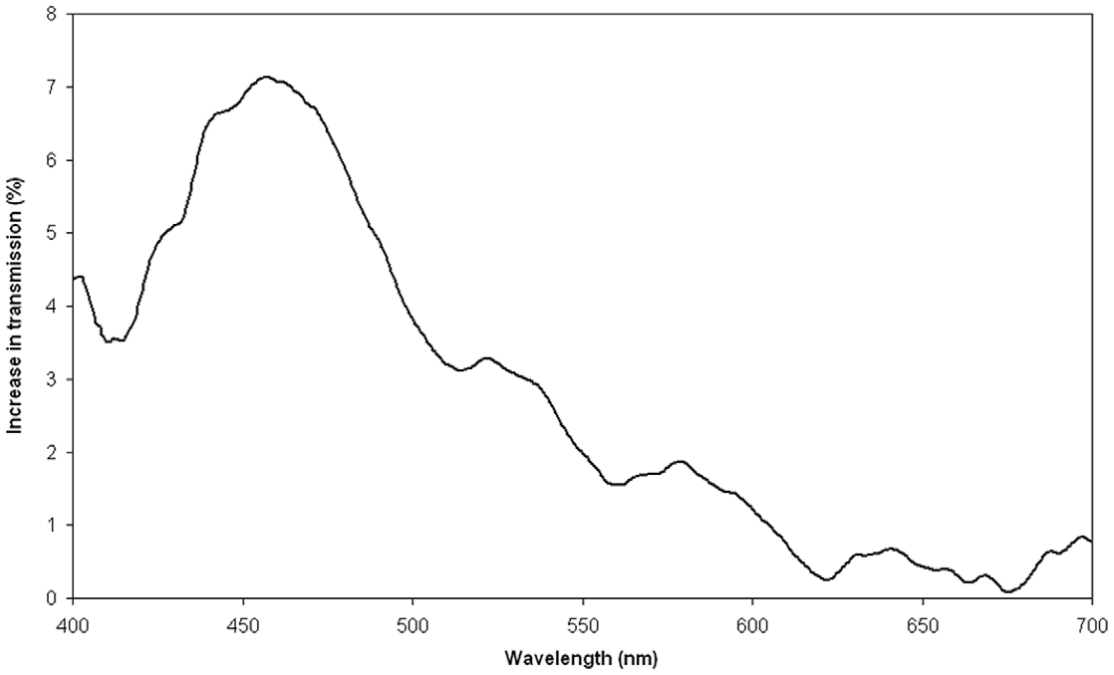
Changes in the transmission properties after treatment. The graph shows the spectral distribution of the increase in light transmission through a human lens (donor lens # 7 in Table 1) after femtosecond laser photolysis. On the y-axis is shown the increase in transmission after treatment. The transmission before treatment was set to 0 for all wavelengths, i.e. 0% indicates that no changes in transmission properties were observed after treatment.

**Figure 3:**
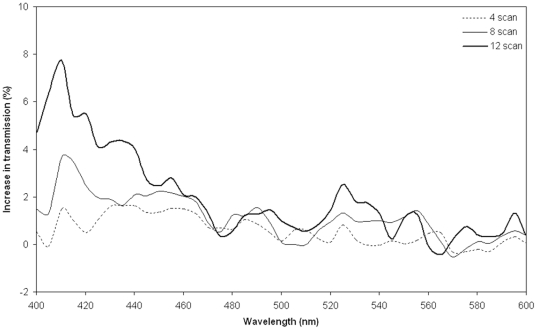
The treatment response depends on the laser dosage. Dose-response curve shows an increasing improvement in the transmission of light with increasing number of scans applied to the same area for a 61 year old human donor lens (donor lens # 6 in Table 1). The increase in transmission is shown in percentage on the y-axis as a function of wavelength (in nm on the x-axis). 0% indicates that no changes were observed after the laser treatment.

After laser treatment the transmission of light through the lens was increased over the entire visible spectrum and most prominently in the blue-green part (Fig. 2), where the age-related loss of transmission is also most pronounced. The effect of the laser treatment showed a clear dose response with the number of scans applied to the lens (Fig. 3).

**Figure 4:**
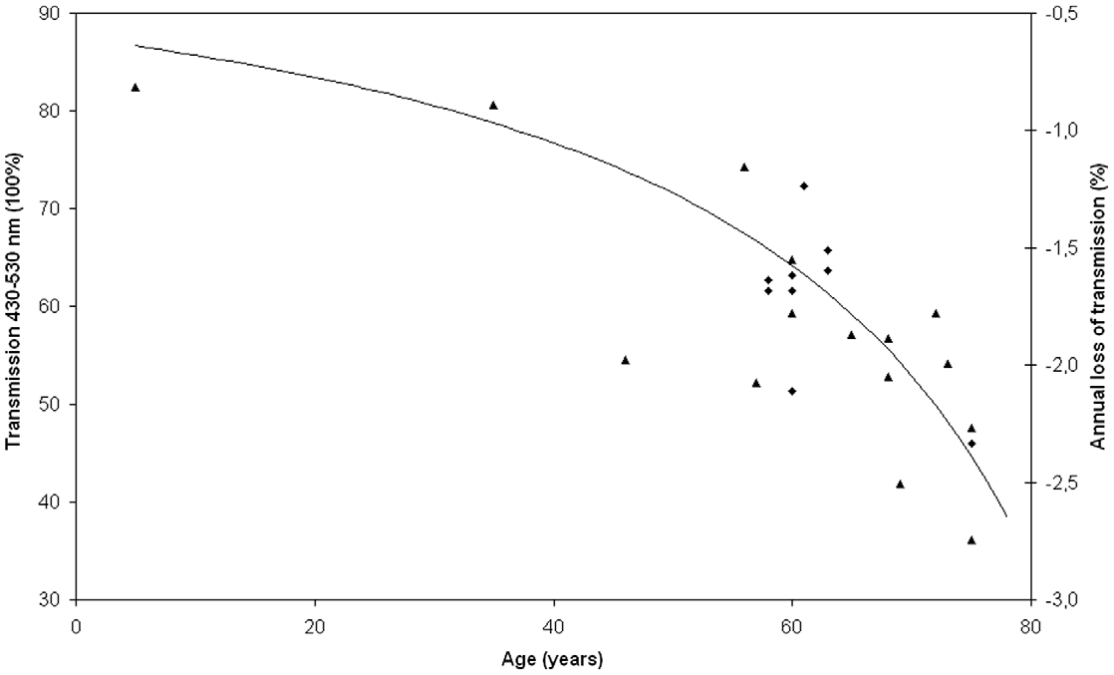
Age-dependent decline in the transmission of blue light. The graph shows the transmission of blue-green light (430–530 nm) measured in vitro in healthy human donor lenses in vitro as a function of age. Diamond markers indicate lenses that were also used in bleaching experiments whereas the triangular marks indicate lenses that were used for transmission measurement only. The left vertical axis shows the transmission from 430 to 530 nm in percent and the right vertical axis show the annual loss of transmission in percent. The curve was drawn based on Eq. 1.

**Figure 5:**
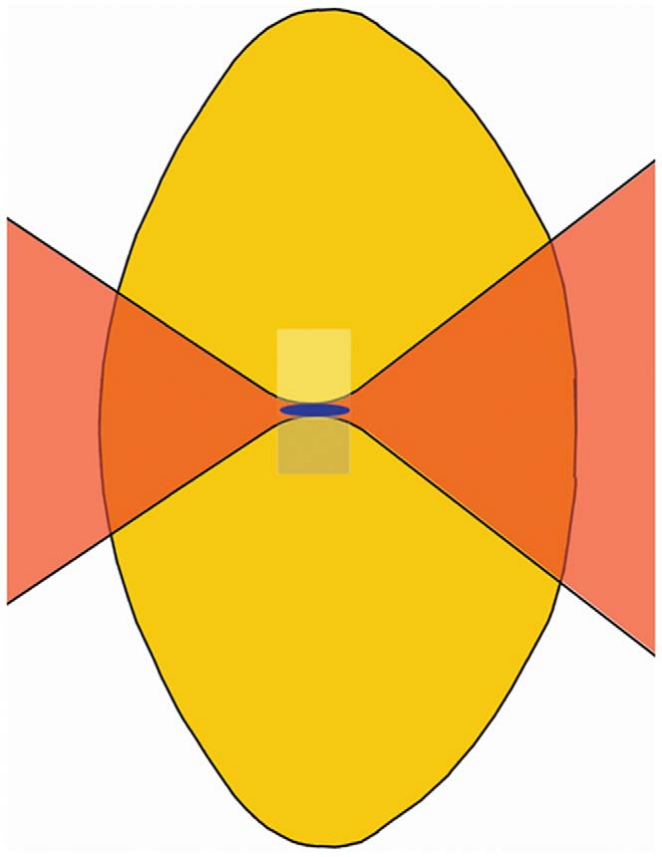
Schematic presentation of the laser treatment shown on a cross section of a lens in the sagittal plane. An infra-red laser (here shown in red) is focused inside a human donor lens. Emission of blue autofluorescence is seen in the focal region as a sign of two-photon absorption. The laser is scanned over the lens in a line-by-line raster pattern. The technical set-up only allowed for 2 directional scanning restricting the depth of the treatment plane to the Rayleigh length of focal region of the laser. Thus, a subvolume measuring 1.0 mm in height by 1.0 mm in width by 0.52 mm in depth was treated. The treated area is shown as a lighter area above the laser focal region and the area awaiting treatment is shown in a grey shade below the laser focal region.

To estimate the clinical relevance of the laser-induced improvement in lens transmission the age-induced changes in lens transmission was assessed in a separate experiment using twenty-five human donor lenses (Fig. 4). We found that the age-related changes in transmission properties of blue-green light from 430 to 530 nm could be approximated by Eq. 1, where *Age* is age in years, p<0.0001, R^2^ = 0.63:
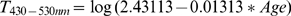
(1)Using Eq. 1 to calculate the apparent lens age after laser treatment, the improvement in lens transmission after treatment was found to correspond to a reduction in lens age of 5.2 years (range 3.0 to 7.3 years, Table 1). Notably, the treatment effects presented in Table 1 was the result of treating a subvolume of the lens of 0.52 mm in thickness, corresponding to 1/10 of the axial thickness of a typical lens (Fig. 5).

**Table 1:** Increase in transmission of human lenses after femtosecond laser photolysis.

Lens no.	Donor age (years)	Transmission increase 430–530 nm (%)	Rejuvenation (years)
1	58	5.4	6.6
2	58	3.5	4.9
3	60	5.9	7.0
4	60	6.0	7.1
5	60	2.1	3.7
6	61	1.3	3.0
7	63	3.1	4.6
8	63	1.6	3.2
**9**	75	6.2	7.3

Rejuvenation was calculated as the difference in age of the donor lens before treatment and the age equivalent to the lens after treatment. Lenses #3 and #4 were from the same donor.

**Figure 6:**
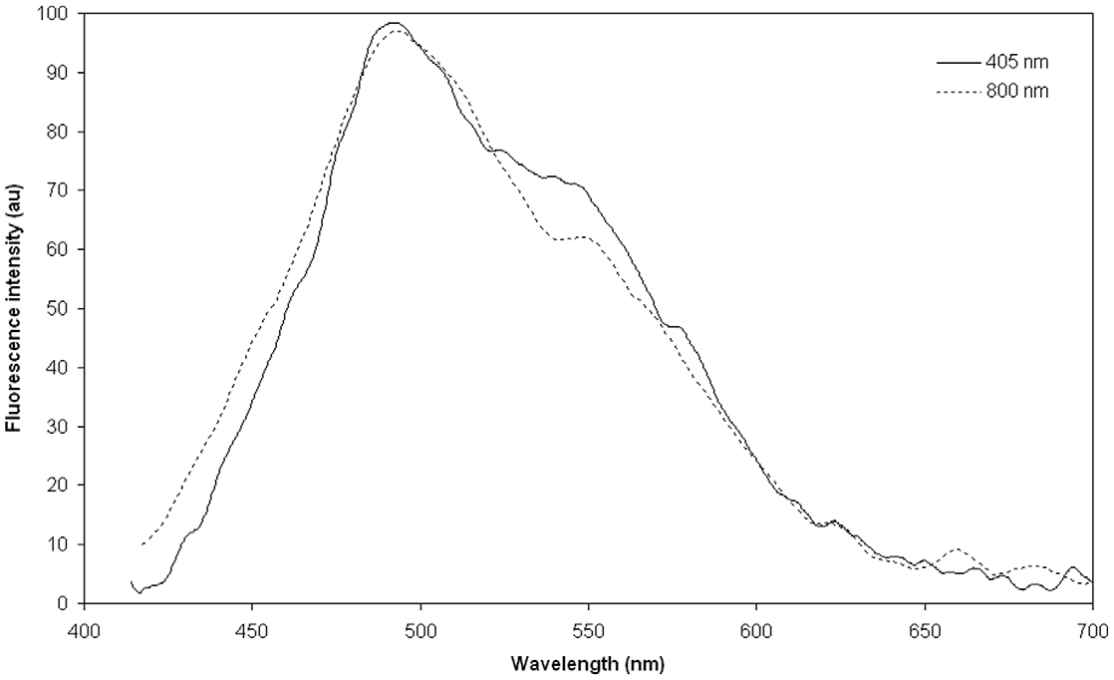
Fluorescence intensity profiles. Spectral characteristics of fluorescence from the human lens excited by 405 nm continuous-wave laser radiation (thin) and 800 nm femtosecond laser radiation (thick). Fluorescence was recorded at a 90 degree angle relative to the excitation laser beam and the differences in fluorescence emission spectrum for λ_ex_ at 405nm and λ_ex_ at 800 nm at the lower wavelengths are related to the 800 nm laser being focused at a greater distance from the detection source, i.e. a greater absorption of preferentially shorter wavelengths took place before the fluorescence emission reached the detector.

During 800 nm femtosecond laser irradiation the lenses emitted a strong blue autofluorescence signal indicating that two-photon absorption took place (Fig. 6). The rate of two-photon absorption (*R_TPA_*)depends on the density (*ρ*) of the molecules that can be excited by two-photon absorption, the two-photon absorption cross section for the molecule (*β*), laser power/area (*I^n^*, with n being the number of photons involved in the process, i.e. n = 2 for a two-photon process), and the energy of the impinging photons (*ℏ︀ω*). Assuming that the medium is optically thin, the rate of TPA of a laser beam impinging on a cylindrical volume with a cross sectional area *A* and length *L* will follow Eq. 2:
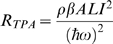
(2)If not only the fluorescence but also the photobleaching was the result of two-photon absorption it follows from Eq. 2 that the rate of photobleaching will be faster in more densely coloured lenses and when more laser power is applied to the lens. In other words, older and more densely coloured lenses will, predictably, benefit more from the laser treatment than younger and less coloured lenses.

We found that, indeed, the photobleaching was significantly (p = 0.01, R^2^ = 0.78) greater for more densely coloured lenses combined with the square of the laser power, Eq. 3:
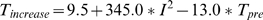
(3)Where *T_increase_* denominates the increase (in percent) in transmission after laser treatment, *T_pre_* denominates the transmission before laser treatment, and *I* is the mean laser power (in Watt). All other variables from Eq. 2 were kept constant between experiments, i.e. the energy of the impinging photons, the area of laser spot size, and the two-photon absorption cross section. The transmission measurements before treatment was used as a surrogate measure of the density of the two-photon absorbing molecules in the lens.

## Discussion

We have shown that the age-induced yellow discolouration of human donor lenses can be bleached by a non-invasive procedure using femtosecond pulsed infra-red laser light. Even with the current primitive set-up clinically relevant effects corresponding to a lens rejuvenation of 3 to 7 years was obtained. Cataract is a disease associated with old age and by postponing the need for surgery by 5 years the number of surgeries performed can be reduced by 35% [19]. Treating the entire lens volume is expected to eliminate the effect of several decades of optical aging.

The observation of blue autofluorescence indicates that the photobleaching was mediated by two-photon absorption of the infra-red laser light leading to ultraviolet equivalent effects in the focal region. Multiphoton effects have a step-like progression with increasing laser intensities from no effects over photobleaching to material ablation and tissue disruption [20]. We used laser intensities well below the level required for mechanical effects [21] because we were interested in the more subtle photochemical effects. The exact photochemical reactions involved in the treatment are not known but ultraviolet irradiation has been shown to degrade one of the advanced glycation end products (AGEs) found in the aging lens [22]. Protein denaturation by advanced glycation is of major importance for the age-induced optical changes seen in human lenses [9] and photodegradation of AGEs could be a likely mechanism of action for the observed photobleaching of the aged human donor lenses.

Despite the widespread use of femtosecond laser in the treatment of medical conditions, most prominently in the LASIK procedure for refractive surgery [23], the ocular safety of multiple femtosecond pulses have only been the subject of a single study so far [24]. Comparing our procedure with this study is not simple because femtosecond retinal safety threshold depends on many different factors, such as retinal spot size [25], pulse duration [26]–[28], and the wavelength of the laser [29]. Nevertheless, based on the existing data our procedure is predicted to within retinal safety limits but further safety evaluation by animal studies should be performed before the treatment can be tested in clinical studies.

Our results are noteworthy because cataract is the most prominent cause of vision loss in the world today. It is responsible for an estimated number of 20 million cases of blindness and an even greater, but unknown, number of cases of impaired visual acuity [1]. Patients are not blinded or visually impaired because of a lack of treatment but because of a lack of access to treatment. A number of economic and logistic challenges are responsible for the lack of access to cataract surgery in underprivileged populations. Changing from an invasive, surgical treatment towards a non-invasive solution will break down some of the barriers impeding access to treatment. If the treatment could be placed in mobile units allowing transportation of the treatment facilities into remote and rural areas, the road is paved for an easy access to a cure for cataract world-wide. Our method has both potentials though many questions must be answered and many issues solved before the treatment can be used clinically, such as the effect of the treatment on the scattering properties of the lens and long term effects of the treatment on the lens as well as the rest of the eye.

In conclusion, our results show that the aged human donor lenses can regain the colour of younger lenses by being treated with subtle low-energy infrared femtosecond laser pulses that evoke two-photon events in a target volume inside the intact lens nucleus. This opens an entirely new prospect of developing clinically applicable non-invasive techniques for the treatment of cataract.

## Materials and Methods

### Human donor lenses

Human lenses were provided by the Cornea Bank NORI (Amsterdam, the Netherlands). Nine lenses from 8 individuals (aged 58–75 years) were used for laser irradiation experiments and these lenses plus 16 additional lenses were included in the study of lens transmission in relation to age (5–73 years). The lenses were maintained at 5°C in minimal essential medium supplemented with phenol red until use. All lenses were of normal yellow color for their age and showed no localized opacities upon microscopy. The quality of the lenses was assessed using slit lamp biomicroscopy and only lenses of good optical quality were used for the experiments. Before the experiments the lenses were removed from the MEM and surgically decapsulated. Then the lenses were sandwiched between two glass microscope mounting plates held apart by an adjustable spacing system. Under these conditions, the lenses maintained their hydration status, transparency and color for up to two weeks post mortem.

### Femtosecond laser system

**Figure 7:**
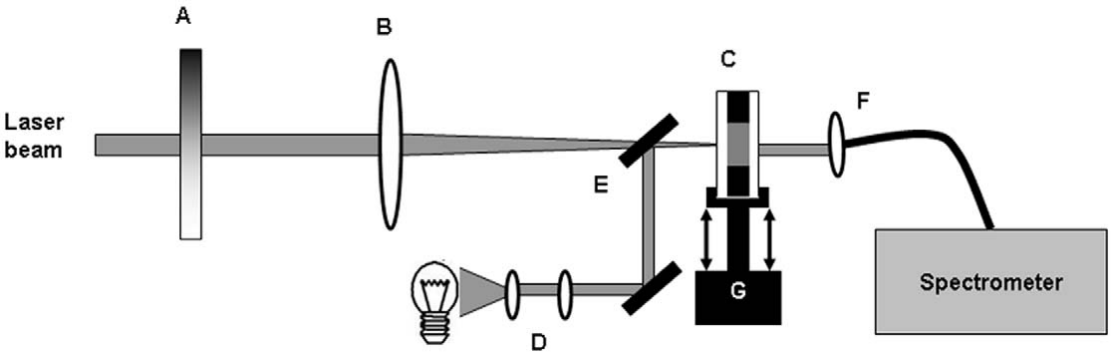
Experimental set-up. Diagram showing the set-up for the experimental laser exposure and transmission study of human lens. Laser intensity was adjusted using a graded neutral density filter (A) and focused using an f = 85 mm focusing lens (B) onto the lens sample (C). Spectral transmission of the lens was measured using an incandescent light source, a set of collimating lenses (D), a flip-in mirror (E), and a spectrometer. The lens sample was moved relative to the laser beam using motorized translation stages (G).

Experimental photolytic treatment of the lens in vitro was performed using a mode-locked Ti:Sapphire laser (Mira 900, Coherent) and a regenerative amplifier (RegA, Coherent). The system delivered a train of pulses with a Gaussian beam profile, the wavelength was centered at 800 nm with maximum pulse energy of 2 µJ and a full-width-at-half-maximum pulse duration of 200 to 300 femtoseconds (10^−15^ s). Repetition rate could be varied from 31 to 275 kHz. Pulse duration and average beam power was measured using an autocorrelator and a thermopile detector, respectively. The laser power delivered to the eye lens was controlled using a graded neutral density attenuation filter. The laser beam was focused using an 85 mm focal length lens yielding a theoretical minimum 1/e^2^ beam intensity diameter of 14 µm and a Rayleigh length of 0.26 mm. This is calculated for the conditions in the lens tissue where the index of refraction is 1.4 [30]. The thickness of the treatment plane is taken to be two times the Rayleigh length, i.e. 0.52 mm. The lens sample was scanned relative to the laser beam using computer-controlled motorized translation stages from Thorlabs (Thorlabs, Sweden). The lens was scanned in a raster pattern in the transverse plane only, meaning that the depth of the treated plane was defined theoretically by the Rayleigh length of the focused laser beam (Fig. 5). The experimental set-up is shown in Fig. 7.

### Transmission measurements

Bleaching was documented by measuring the transmission of white light from an incandescent light bulb source that was collected by a spectrometer (AvaSpec-2048-2 spectrometer (Avantes, the Netherlands). To ensure that transmission was measured at the exact site of exposure, the transmission measurements were aligned with the laser exposure beam using a 100 µm (diameter) aperture. To correct for minor differences in intensity of the white light source, all transmission measurements shown have been calibrated to the transmission of near-infrared light (750–800 nm).Though measurements of scattering of light was not directly included in the study, indirect information on scattering properties can be derived from the transmission measurements since an increase in scattering (especially backward scattering, i.e. in the direction backwards to the incandescent light source) would reduce the recorded transmission.

### Slit lamp biomicroscopy

The lenses were inspected visually before and after treatment using a slit lamp (BQ900, Haag-Streit, Germany). The slit lamp is a modified microscope where the object of interest can be viewed at 6.3 to 40× magnification using a height and width adjustable slit of light that illuminates the object of interest at a chosen angle, thus providing the possibility three-dimensional optical sectioning.

### Photographic documentation

The lenses were photographed using a digital Canon 30D EOS camera equipped with a macrolens.
